# Case Report: A rare case of tuberculous otitis media mimicking chronic suppurative otitis media — an ongoing challenge

**DOI:** 10.3389/fmed.2025.1521011

**Published:** 2025-07-14

**Authors:** Chengjie Shu, Taixian You, Mei Huang, Minglong Xu, Jianyong Zhang, Zhangli Peng

**Affiliations:** ^1^Tuberculosis Division of Pulmonary and Critical Care Medicine, Affiliated Hospital of Zunyi Medical University, Zunyi, Guizhou, China; ^2^Department of General Practice, Affiliated Hospital of Zunyi Medical University, Zunyi, China

**Keywords:** middle ear tuberculosis, facial paralysis, *Mycobacterium tuberculosis*, hearing loss, otorrhea

## Abstract

Ear tuberculosis, a rare form of extrapulmonary tuberculosis caused by *Mycobacterium tuberculosis*, predominantly affects the middle ear. The common symptoms of ear tuberculosis include otorrhea, hearing loss, and facial nerve paralysis, and if left untreated, it can lead to complete deafness. Diagnosing ear tuberculosis can be challenging, as its symptoms often overlap with those of otitis media, leading to potential misdiagnosis. Early diagnosis and appropriate treatment are essential for a favorable prognosis. Delayed diagnosis or inadequate treatment can result in severe complications, including irreversible hearing loss and chronic ear problems. Therefore, raising awareness among healthcare providers about the clinical features and diagnostic approach to ear tuberculosis is critical for improving patient outcomes. This study presents the case of a 28-year-old patient with tuberculous otitis media (TOM), presenting with otorrhea, hearing loss, and facial paralysis. Additionally, a comprehensive literature review of 492 records published over the past decade in PubMed and the Web of Science databases was conducted. Our study summarizes the clinical manifestations, diagnostic methods, and treatment strategies of 118 patients with ear tuberculosis, offering valuable insights to support early diagnosis and intervention, ultimately reducing the risk of adverse outcomes.

## Introduction

Tuberculosis (TB) is an infectious disease caused by *Mycobacterium tuberculosis* (Mtb). Although it primarily affects the lungs (pulmonary tuberculosis), it can also involve other parts of the body, resulting in extrapulmonary tuberculosis (1). Ear tuberculosis is a form of extrapulmonary TB caused by Mtb. It is a rare condition, accounting for approximately 0.1% of all TB cases (2–4) and 0.04–0.9% of chronic suppurative otitis media (5, 6). It can affect the external ear canal, middle ear, and inner ear, with the middle ear being the most commonly involved site. Typical features of tuberculous otitis media (TOM) include a perforated eardrum and the presence of granulation tissue (7–9). Although the incidence of TOM is extremely low, it can lead to persistent ear discharge, significant hearing loss, and even facial paralysis, severely impacting patients’ health and quality of life (10–12). The non-specific clinical manifestations of ear tuberculosis often mimic other ear infections, leading to misdiagnosis and delayed treatment. Therefore, a comprehensive understanding of the clinical features and diagnostic approaches for ear tuberculosis is essential for the timely identification and effective management of this rare condition.

### Case report

**Figure 1:**
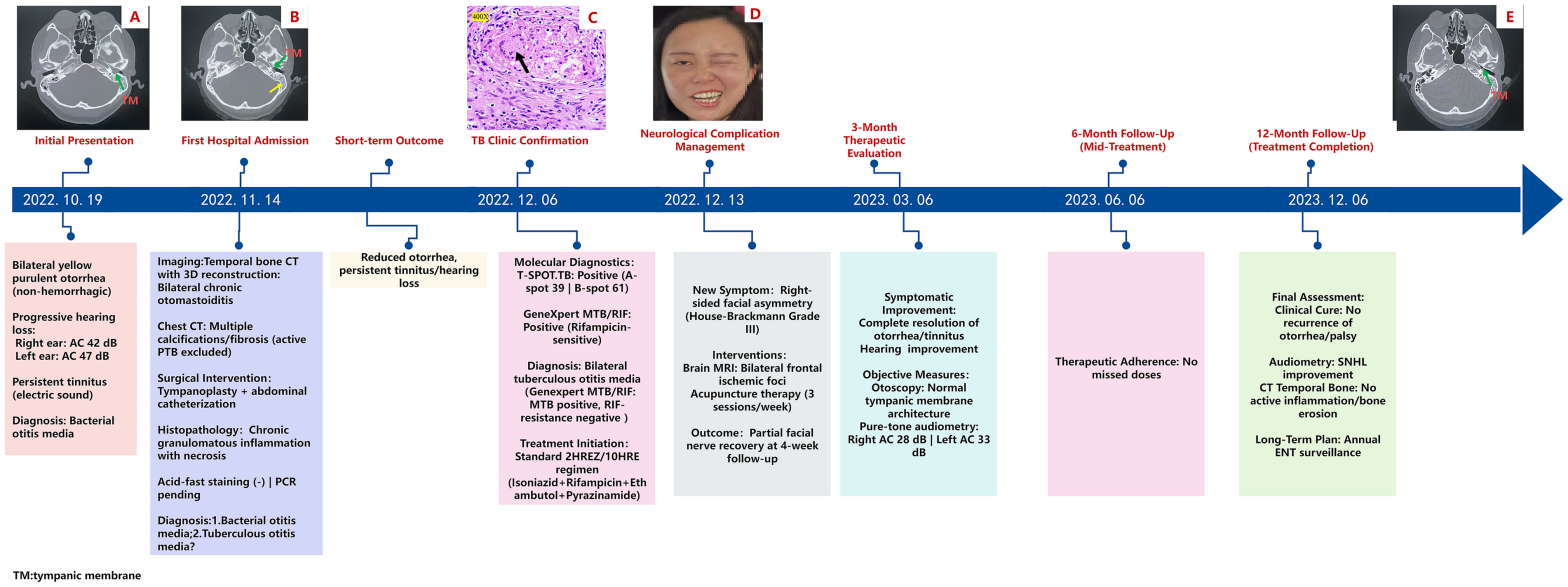
Clinical timeline of diagnosis, treatment, and follow-up in a case of tuberculous otitis media (TOM) with facial nerve paralysis: **(A)** Axial bone-window computed tomography (CT) scan of the temporal bone shows a connection between the left tympanic cavity and the external auditory canal, with increased density in the mastoid process. The green arrow indicates the tympanic membrane (TM) perforation. **(B)** Axial bone-window CT scan of the temporal bone demonstrates a left tympanic membrane (TM) perforation, accompanied by fluid opacification in the ipsilateral mastoid air cells. The green arrow points to the perforation of the tympanic membrane, while the yellow arrow highlights fluid accumulation in the mastoid. **(C)** Necrotic tissue resected from the left tympanic cavity, stained with hematoxylin and eosin (HE), reveals necrotizing granulomas (black arrowheads) at 400x magnification. **(D)** Right-sided peripheral facial paralysis. **(E)** Follow-up at 12 months: Axial bone-window CT scan of the temporal bone shows an intact left tympanic membrane (TM) and reduced opacification in the ipsilateral mastoid air cells.

A 28-year-old female presented with a 2-month history of bilateral otorrhea, characterized by purulent discharge, followed by progressive conductive hearing loss over the preceding week. The diagnostic and therapeutic workflow is depicted in Figure 1, highlighting the key clinical decisions and interventions. The patient first noticed yellowish mucopurulent otorrhea on 19 October 2022, with no apparent cause or presence of blood. This was followed by subjective hearing impairment and persistent tinnitus (described as an electric sound). Occasionally, she also experienced a sensation of ear swelling but had no fever or ear pain. She was evaluated as an outpatient at the Ear, Nose, and Throat (ENT) clinic of our hospital.

On examination, bilateral nasal mucosal chronic congestion and swelling, along with bilateral inferior turbinate hypertrophy, were noted. No secretions were observed in the middle or lower nasal passages, and the nasal septum was straight. The external ear canals were patent, and tympanic membrane retraction was noted. A computed tomography (CT) scan with 3D reconstruction (Figure 1A) of the temporal bone showed bilateral chronic middle ear infection and mastoiditis, with possible cholesteatoma on the left side (magnetic resonance imaging (MRI) was recommended). The scan also revealed thickened bilateral maxillary sinus mucosa and hypertrophy of the bilateral inferior turbinates. Pure-tone audiometry results revealed the following results: in the right ear, air conduction (AC) was 42 dB and bone conduction (BC) was 8 dB; and in the left ear, AC was 47 dB and BC was 7 dB. The patient was diagnosed with “bilateral chronic suppurative otitis media” and initially treated with anti-inflammatory medications, which improved the purulent discharge but did not alleviate her tinnitus or hearing loss.

On 14 November 2022, the patient was admitted to the ENT department for surgical treatment. Further examinations upon admission revealed no abnormalities in routine blood tests, liver function, kidney function, coagulation profile, and stool analysis. An axial bone-window CT scan of the temporal bone confirmed a perforated left tympanic membrane (TM) (Figure 1B). Pure-tone audiometry confirmed bilateral hearing loss. Chest CT revealed bilateral emphysema, multiple calcifications and fibrosis in both lungs, and pleural thickening. Active pulmonary tuberculosis was excluded. After departmental consultation, tympanoplasty and tympanic catheterization were performed. Pathological examination from the left ear indicated chronic granulomatous inflammatory necrosis, with a recommendation for acid-fast staining to aid in diagnosis. A subsequent pathological report of the left ear confirmed chronic granulomatous inflammatory necrosis. Clinical and laboratory tests were recommended to rule out tuberculosis and other granulomatous diseases (Figure 1C). Special staining results were negative for acid-fast bacilli. Based on the available findings, a provisional diagnosis of bacterial or tuberculous otitis media was considered. Following surgery, the patient was treated symptomatically and discharged once her general symptoms improved, although tinnitus and hearing loss persisted.

On 1 December 2022, during a follow-up visit at the outpatient clinic, a physical examination of the patient revealed secretion in the left ear canal. Otoscopic examination showed tympanic membrane perforation, hyperemia of the surrounding mucosal tissue, and a small amount of purulent secretion. Histopathological examination revealed chronic granulomatous inflammation, with tuberculosis still being a possibility. The patient was referred to the tuberculosis clinic, where further diagnostic tests were conducted. A T-SPOT. TB test was performed and the results were positive (+), with 39 A-hole spots and 61 B-hole spots. The GeneXpert test (*M. tuberculosis* nucleic acid test) was also positive (+), but the rifampicin resistance gene was negative. Mycobacterial culture was negative. Based on these findings, the patient was diagnosed with bilateral tuberculous otitis media (Genexpert MTB/RIF: MTB positive, Rifampicin (RIF-resistance) negative). Treatment with a 2 Isoniazid/Rifampicin/Ethambutol/Pyrazinamide/10 Isoniazid/Rifampicin (2HREZ/10HR) anti-tuberculosis regimen was initiated on 6 December 2022.

On 13 December 2022, the patient presented with a crooked mouth angle and was admitted to the Department of Neurology. Physical examination revealed shallow right frontal striae, incomplete closure of the right ophthalmic fissure, a shallow right nasolabial groove, and air leakage during the cheek puffing test (Figure 1D). A head MRI plain scan showed bilateral frontal ischemia. The patient was diagnosed with “peripheral facial nerve paralysis” and was recommended acupuncture treatment, in addition to continuing the anti-tuberculosis therapy.

The patient underwent scheduled monthly follow-up evaluations. At the 3-month post-therapy milestone (6 March 2023), a comprehensive post-treatment evaluation was conducted. Otoscopic examination revealed an intact tympanic membrane, and pure-tone audiometry revealed mild bilateral hearing loss (air conduction thresholds: right ear - 28 dB, left ear - 33 dB; normal range: ≤25 dB). The subjective symptoms of otorrhea and tinnitus had completely resolved, and her hearing had partially improved. The patient continued with the anti-tuberculosis regimen (9HR).

**Figure 2:**
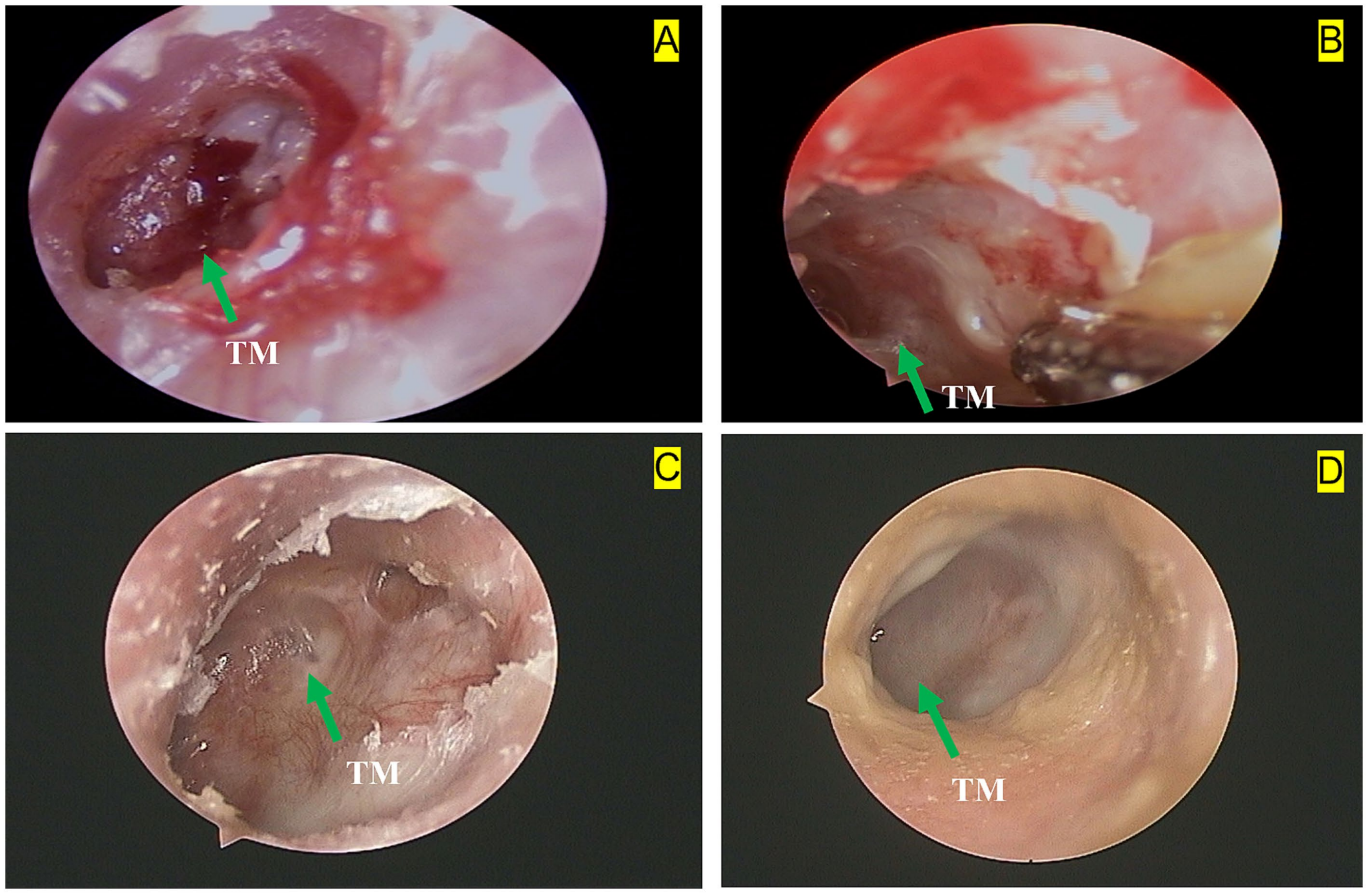
Left otoscopic dynamic changes in a 28-year-old female with tuberculous otitis media (TOM): **(A)** Baseline otoscopy shows a tympanic membrane (TM) perforation, hyperemia of the surrounding mucosal tissue, and a small amount of purulent discharge. The green arrow indicates the tympanic membrane perforation. **(B)** Three months after treatment with anti-tuberculosis drugs and surgery, otoscopy reveals a small amount of purulent discharge and persistent hyperemia in the surrounding mucosal tissue. The green arrow highlights the tympanic membrane (TM). **(C)** Six months after treatment withdrawal, otoscopy shows an intact tympanic membrane (TM) with hyperplastic scar tissue at the edges. The green arrow points to the tympanic membrane. **(D)** Twelve months after treatment withdrawal, otoscopy shows an intact tympanic membrane (TM) with smooth, healthy peripheral mucosa. The green arrow indicates the tympanic membrane.

On 6 December 2023, an axial bone-window CT scan of the temporal bone showed an intact left tympanic membrane (Figure 1E), leading to the cessation of anti-tuberculosis treatment. Initial otoscopy at this point revealed tympanic membrane perforation, mucosal hyperemia, and purulent secretions (Figure 2A). After 3 months of standard anti-tuberculosis treatment, the tympanic membrane had healed, and no residual discharge was present (Figure 2B). Follow-up otoscopic evaluations conducted at 6 months (Figure 2C) and 12 months (Figure 2D) after treatment cessation revealed a persistently intact tympanic membrane, with no signs of further infection or complications.

Previous medical history: the patient was diagnosed with Henoch–Schönlein purpura nephritis approximately 20 years ago and was treated with corticosteroids for 1 year. A recurrence occurred 7 years ago, requiring another 1-year course of corticosteroid therapy. She also experienced recurrent nasopharyngeal discomfort. Nasopharyngoscopy revealed chronic pharyngitis and sinusitis, and she received intermittent anti-infective treatment for 3 months.

Personal history: the patient had received the Bacillus Calmette–Guérin (BCG) vaccination in childhood. There were no known risk factors for tuberculosis exposure, such as living in an endemic area or having close contact with a confirmed TB case.

## Literature review

### Methods

**Figure 3:**
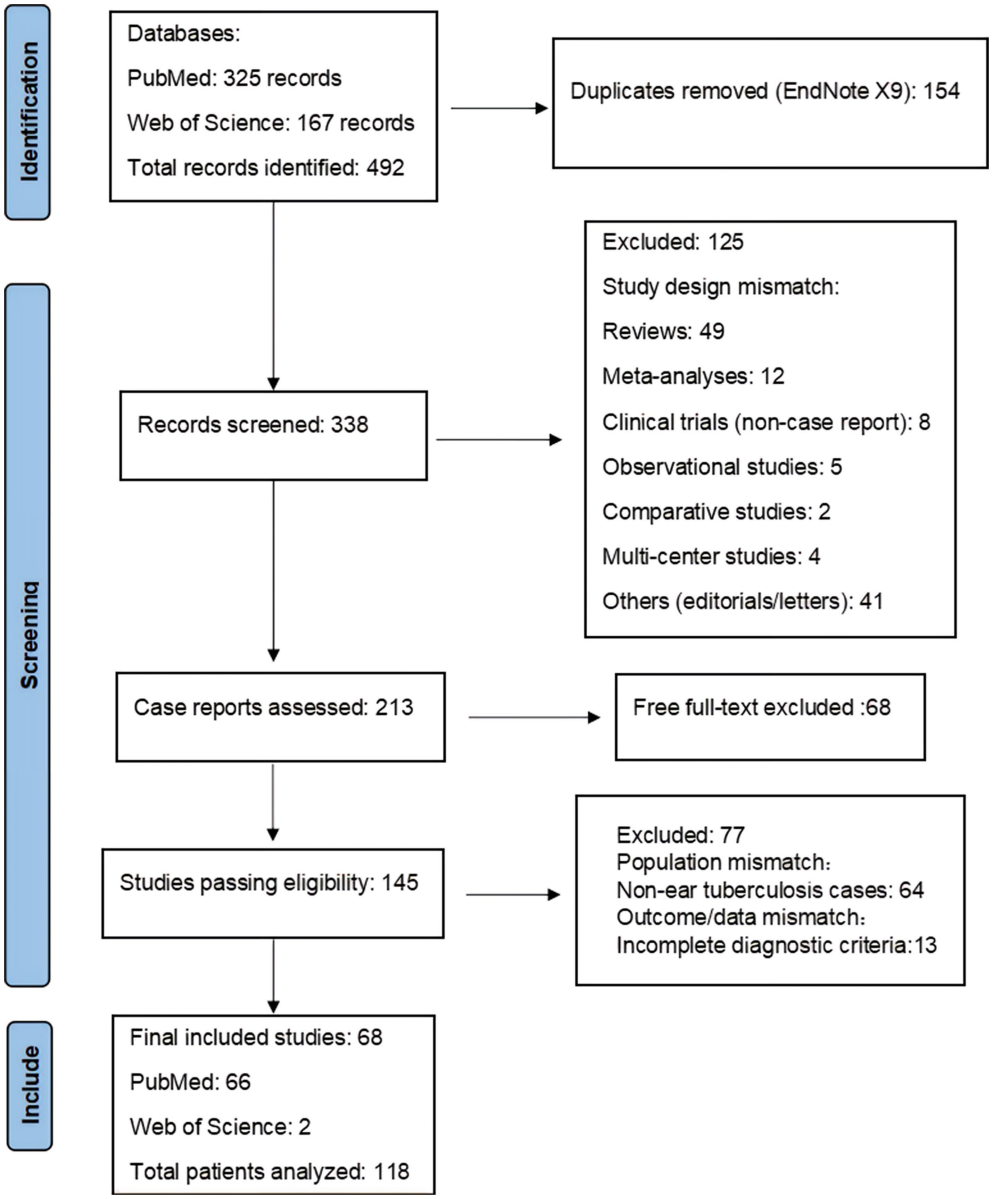
Graphical representation of the literature review search process based on Page et al. (32).

A systematic literature search was conducted in PubMed for the period from January 2014 to December 2023 using the Boolean search terms “(tuberculous otitis) OR (tuberculosis of ear),” which identified 325 records. Additionally, a search in the Web of Science database using the terms “TS = (tuberculosis OR ‘*mycobacterium tuberculosis*’ OR tuberculous) AND TS = (ear OR auricular OR otitis OR ‘middle ear’ OR ‘external ear’ OR mastoid OR ‘temporal bone)” yielded 167 records. In total, 492 records were identified across both databases over the past decade. Only articles that met the specific criteria for tuberculous otitis were included in the analysis. Ultimately, 68 unique case reports were included (PRISMA flow diagram, Figure 3), representing 118 patients from 24 countries. A summary of the literature reviewed and the key findings are provided in Supplementary Table S1.

## Results

### Tuberculous otitis media in young and middle-aged patients: no obvious gender association

Tuberculous otitis media primarily affects young and middle-aged individuals, with no significant gender bias. While pulmonary tuberculosis tends to more frequently affect younger male individuals, the incidence of tuberculous otitis media does not show any gender differences. Of the 114 patients with recorded gender data, 53 (46.49%, 53/114) were male and 61 (53.51%, 61/114) were female, with 4 cases lacking gender information. No statistically significant gender difference was observed. In terms of age distribution, the cases were as follows: 0–17 years: 38 cases (32.48%, 38/117), 18–34 years: 25 cases (21.37%, 25/117), 35–64 years: 42 cases (35.90%, 42/117), and above 65 years: 12 cases (10.25%, 12/117). One case did not specify the exact age. These findings indicate that tuberculous otitis media predominantly affects younger and middle-aged individuals.

### Primary tuberculous otitis media was more common than secondary tuberculous otitis media

Extrapulmonary tuberculosis is often secondary to pulmonary tuberculosis (PTB). Among the 118 patients with ear tuberculosis, 76.27% (90/118) had isolated ear tuberculosis, while 23.72% (28/118) had concurrent tuberculosis in other sites, including pulmonary tuberculosis. Among the 28 patients with concurrent tuberculosis, 17 (60.71%, 17/28) had pulmonary tuberculosis (PTB), 4 (14.28%, 4/28) had nasopharyngeal tuberculosis, 3 (10.71%, 3/28) had both PTB and meningoencephalitis, 2 (7.14%, 2/28) had both PTB and spinal tuberculosis, 1 (3.57%, 1/28) had cervical tuberculous lymphadenitis, and 1 (3.57%, 1/28) had abdominal tuberculosis (detailed in Supplementary Table S2).

### Otorrhea and hearing loss, are typical symptoms of tuberculous otitis media

**Figure 4:**
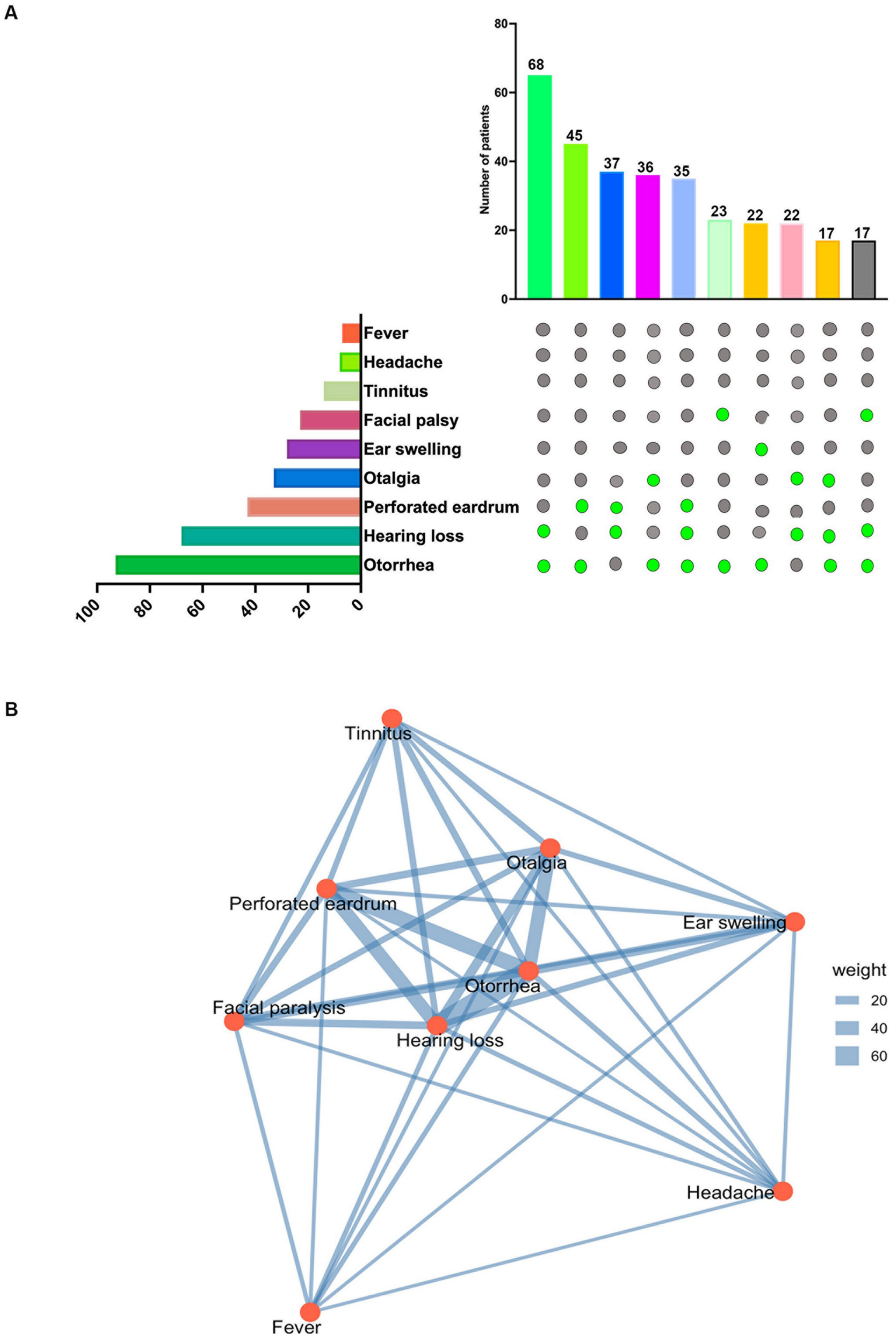
Symptom prevalence and interrelationships: **(A)** Symptom prevalence: the upper graph displays the top 10 symptom combinations observed in 118 patients. The lower graph shows the total number of patients experiencing each symptom among the 118 individuals with tuberculous otitis media (TOM). **(B)** Symptom network: a network graph generated using RStudio and the igraph package illustrates the relationships between symptoms. Lines connecting symptom nodes represent conditional dependencies, with thicker lines indicating stronger positive conditional associations.

The clinical presentation of ear tuberculosis varied across the 118 patients, with an average disease duration of 18.2 months. The most common symptoms were otorrhea (78.81, 95% Confidence Interval (CI): 70.82–85.07%), followed by hearing loss (57.63, 95% CI: 48.63–66.18%) and perforated eardrum (36.44, 95% CI: 28.26–45.39%) (Figure 4A). The classical triad of tuberculous otitis media consists of otorrhea, hearing loss, and tympanic membrane perforation, and was observed in 29.66% of cases (35/118). In contrast, the dyad of hearing loss and otorrhea was more commonly observed, occurring in 57.63% of patients (68/118). To explore the relationships between these symptoms, we utilized RStudio with the ‘igraph’ and ‘corrplot’ packages. The majority of symptoms exhibited conditional dependence with at least one other symptom. In the network symptom graph (Figure 4B), hearing loss emerged as a central node, showing strong association with otorrhea and perforated eardrum. We calculated the Pearson correlation coefficient using RStudio with the corrplot package to quantify these associations. Supplementary Figure S1 shows that the absolute value of the Pearson correlation coefficient is greater than 0.2, with a *p*-value of <0.05, indicating a positive correlation between hearing loss and perforated eardrum (*r* = 0.29).

### Temporal CT scan and PCR testing improve the diagnosis rate of tuberculous otitis media

Diagnosing ear tuberculosis requires the use of multiple diagnostic methods. Laboratory examinations play a critical role in confirming the diagnosis. Among the cases reviewed, histopathology was positive in 84 cases (89.36%, 84/94), acid-fast staining was positive in 55 cases (64.70%, 55/85), and polymerase chain reaction (PCR) testing was positive in 49 cases (81.67%, 49/60). Additionally, temporal bone CT scans showed bone destruction in 40 cases (86.96%, 40/46). Mycobacterial cultures were positive in 24 cases (60%, 24/40), while 3 cases were diagnosed based on effective responses to anti-tuberculosis therapy (detailed in Supplementary Table S3). The average diagnostic delay was 11.5 months. In conclusion, early PCR testing of local secretions and temporal bone CT scans can significantly enhance the diagnostic rate of tuberculous otitis media.

### Standard HRZE treatment ensures favorable prognosis in tuberculous otitis media

Patients diagnosed with tuberculous otitis media typically undergo standard anti-tuberculosis therapy, often accompanied by adjuvant surgery. Among the 118 patients in our review, 43.2% (51/118) received the HRZE (isoniazid, rifampicin, pyrazinamide, and ethambutol) regimen, and 92.2% (47/51) of these patients showed a favorable prognosis. Additionally, 5.1% (6/118) of patients received the HRZ regimen, with one patient exhibiting poor outcomes and no significant symptom improvement. Two patients with drug-resistant TB strains were treated with a combination regimen of linezolid, moxifloxacin or linezolid, cycloserine, prothionamide, pyrazinamide, ethambutol, and bedaquiline, and both patients had a good prognosis.

Surgical intervention was performed in 50.0% (59/118) of cases, including tympanoplasty, mastoidectomy, facial nerve decompression, and myringotomy. Of these cases, 57 patients began anti-tuberculosis therapy immediately after the pathology confirmed the diagnosis of tuberculous otitis, while two patients underwent surgery after 8 months of anti-tuberculosis therapy. Surgery improved symptoms in these patients. Following treatment, 89.8% (106/118) of patients experienced symptom improvement and a favorable prognosis. However, in 8 patients, the symptoms were either stable or worsened, while in 4 patients, the outcomes were unknown.

## Discussion

Ear tuberculosis is a form of extrapulmonary tuberculosis that predominantly affects the middle ear. It is a rare condition, accounting for approximately 0.1% of all tuberculosis cases and 0.04–0.9% of chronic suppurative otitis media cases. Due to the non-specific clinical symptoms of middle ear tuberculosis, obtaining reliable bacteriological confirmation can be challenging, which contributes to a reduced clinical awareness of the disease (13).

Previous studies have suggested that patients with extrapulmonary tuberculosis often have concurrent pulmonary tuberculosis (14, 15). However, the data from this literature review indicate that 76.27% of patients had isolated primary ear tuberculosis, which aligns with other reports. As an example, Rajiv C. Michael et al. reported that the majority of their patients had primary ear tuberculosis (16), which could be attributed to the complex anatomy and pathogenesis of the ear. The pathogenesis of tuberculous otitis media (TOM) involves two primary mechanisms: first, the middle ear serves as the primary site of infection, with mycobacteria spreading directly through the Eustachian tube, external auditory canal, or perforated tympanic membrane; and second, the middle ear is secondarily infected via hematogenous or lymphatic spread, or through the Eustachian tube (6). In our patient, the first mechanism may apply, as the infection likely originated in the middle ear. Notably, our patient also had a history of Henoch–Schönlein purpura nephritis and received long-term corticosteroid therapy, suggesting that the route of infection in extrapulmonary tuberculosis may not necessarily involve intrapulmonary spread but could also be influenced by the host’s immune status (3).

Ear tuberculosis, though rare, has been recognized as a serious condition for decades. In 1953, Wallmer was the first to describe the clinical manifestations of tuberculous otitis media (3, 17). Due to its atypical symptoms, TOM is often misdiagnosed. Our data revealed a variety of clinical manifestations in ear tuberculosis, including otorrhea (78.81%), hearing loss (57.63%), tympanic membrane perforation (36.44%), facial palsy (19.5%), headache (6.7%), and fever (5.9%). The classic triad of otorrhea, hearing loss, and tympanic membrane perforation remained the most prevalent combination, accounting for 29.66% of cases. In our case, this triad was present along with facial nerve palsy, which is consistent with the findings reported by Ki-Eun Hwang (6). It has been reported that 15–40% of patients with middle ear tuberculosis may experience facial paralysis (18). Patients with immune system abnormalities can experience a more severe course of ear tuberculosis, and those with immunodeficiency are at increased risk of severe symptoms and complications, such as deafness and facial nerve paralysis. Although our patient had a history of renal purpura, the absence of further immunological assessment (e.g., CD4/CD8 profiling) limits our ability to completely evaluate her immune status.

The diagnosis of ear tuberculosis typically relies on laboratory tests (19). Smear microscopy can detect acid-fast bacilli (AFB) in ear secretions, and the culture method can confirm the presence of *Mycobacterium tuberculosis* while also assessing antibiotic sensitivity. However, the culture method is time-consuming and not ideal for early diagnosis. Histological examination of ear tissue is crucial, with necrotizing granulomas being a characteristic feature (20). PCR techniques, including Genexpert. TB, enable rapid detection of *M. tuberculosis* DNA and are valuable for early diagnosis and drug resistance screening. This method is quick, accurate, and provides reliable results (2, 21). In 2004, PCR-reverse hybridization was reported as a useful tool for diagnosing tuberculous otitis media (TOM) (22). In 2015, fine-needle aspiration cytology (FNAC) was introduced as a diagnostic tool for pediatric head and neck lymphadenopathy (23). Given the non-specific clinical manifestations of ear tuberculosis, early diagnosis remains challenging (9). Our literature review showed positivity rates of 86.96, 89.36, 64.7, 81.67, and 60% for temporal bone CT, histopathological examination, acid-fast staining, PCR tests, and mycobacterial culture, respectively. These findings suggest that histopathological examination, temporal CT, and PCR tests significantly enhance diagnostic accuracy. Kriukov et al. reported that the diagnosis of TOM was achieved via AFB staining (9%), cytology (27.3%), histopathology (18%), and PCR (55%) (24). Similarly, Susan et al. found that histopathology and AFB staining of middle ear tissue were positive in 90% of cases (25). Although mycobacterial culture remains the gold standard for diagnosis, in cases with negative etiological tests, temporal bone CT scanning and pathological examination can serve as valuable supplementary tools for diagnosis.

Once the condition is diagnosed, anti-TB treatment should be promptly initiated. Standard anti-TB medications include isoniazid, rifampicin, pyrazinamide, and ethambutol. Tuberculosis typically follows a prolonged course, and treatment progresses slowly. However, adherence to the prescribed regimen and dosage schedule is essential for effective treatment. In severe cases of middle ear tuberculosis, or when the condition does not adequately respond to medication, surgical intervention may be necessary. Surgical options include mastoidectomy, tympanoplasty, tympanostomy tube insertion, and subtotal temporal bone resection. The primary goal of these procedures is to remove pathological tissue from the affected ear, reducing inflammation and restoring hearing function. In some cases, additional surgeries, such as facial nerve decompression, may be required (13, 26, 27). Adjuvant therapy is also necessary, particularly in cases of facial nerve paralysis or hearing loss. Studies by Zhidan Wang et al. suggest that acupuncture treatment can improve facial paralysis symptoms by reducing edema in the acute stage, aiding facial blood circulation, and enhancing muscle activity (26, 28). Similar findings have been reported in studies by Cui H et al. and Cheng L (29, 30). In patients with severe hearing loss, cochlear implants can be considered to improve hearing (31). In alignment with these findings, our patient received adjuvant therapy, including acupuncture and surgical treatment, along with continued anti-tuberculosis pharmacotherapy. As a result, all symptoms resolved, and follow-up otoscopy showed normalization of the affected ear.

This case underscores the diagnostic challenges and clinical implications of ear tuberculosis. The patient’s initial presentation with chronic otorrhea and hearing loss, coupled with inconclusive histopathological findings, highlights the difficulty in distinguishing tuberculous otitis media from other chronic infectious and inflammatory conditions. The eventual confirmation of *Mycobacterium tuberculosis* through molecular diagnostics emphasizes the importance of considering TOM in patients with persistent otologic symptoms, hearing loss, and facial palsy, particularly in endemic regions. Additionally, the development of peripheral facial nerve paralysis suggests potential neurological involvement, underscoring the need for a multidisciplinary approach to managing TOM. This case emphasizes the need for heightened clinical awareness, early molecular testing, and integrated treatment strategies to improve patient outcomes.

## Conclusion

Tuberculous otitis media is a rare condition that poses diagnostic challenges due to limited clinical awareness. In patients with persistent symptoms that do not improve, thorough etiological and histopathological investigations are critical for effective management. Early diagnosis is key to effective treatment, and timely initiation of anti-TB therapy can significantly improve patient outcomes, as demonstrated in this case. While surgery may help alleviate symptoms, the risk of recurrence highlights the importance of early diagnosis and timely intervention in ear tuberculosis.

## Data Availability

The original contributions presented in the study are included in the article/Supplementary material, further inquiries can be directed to the corresponding authors.

## Supplementary material

SUPPLEMENTARY FIGURE S1 Matrix of Pearson’s correlation coefficients among different symptoms: A color-coded correlation scale is displayed to the right of the plot. Blue represents positive correlations, while red ellipses indicate negative correlations. This heatmap includes correlations with an absolute Pearson correlation coefficient greater than 0.2 and a *p*-value < 0.05.
