# Supplementary figures and images for: Case Report: A rare case of tuberculous otitis media mimicking chronic suppurative otitis media — an ongoing challenge
Source: Front Med (Lausanne). 2025 Jul 14;12:1521011. doi: 10.3389/fmed.2025.1521011 (PMC12301375; doi:10.3389/fmed.2025.1521011)

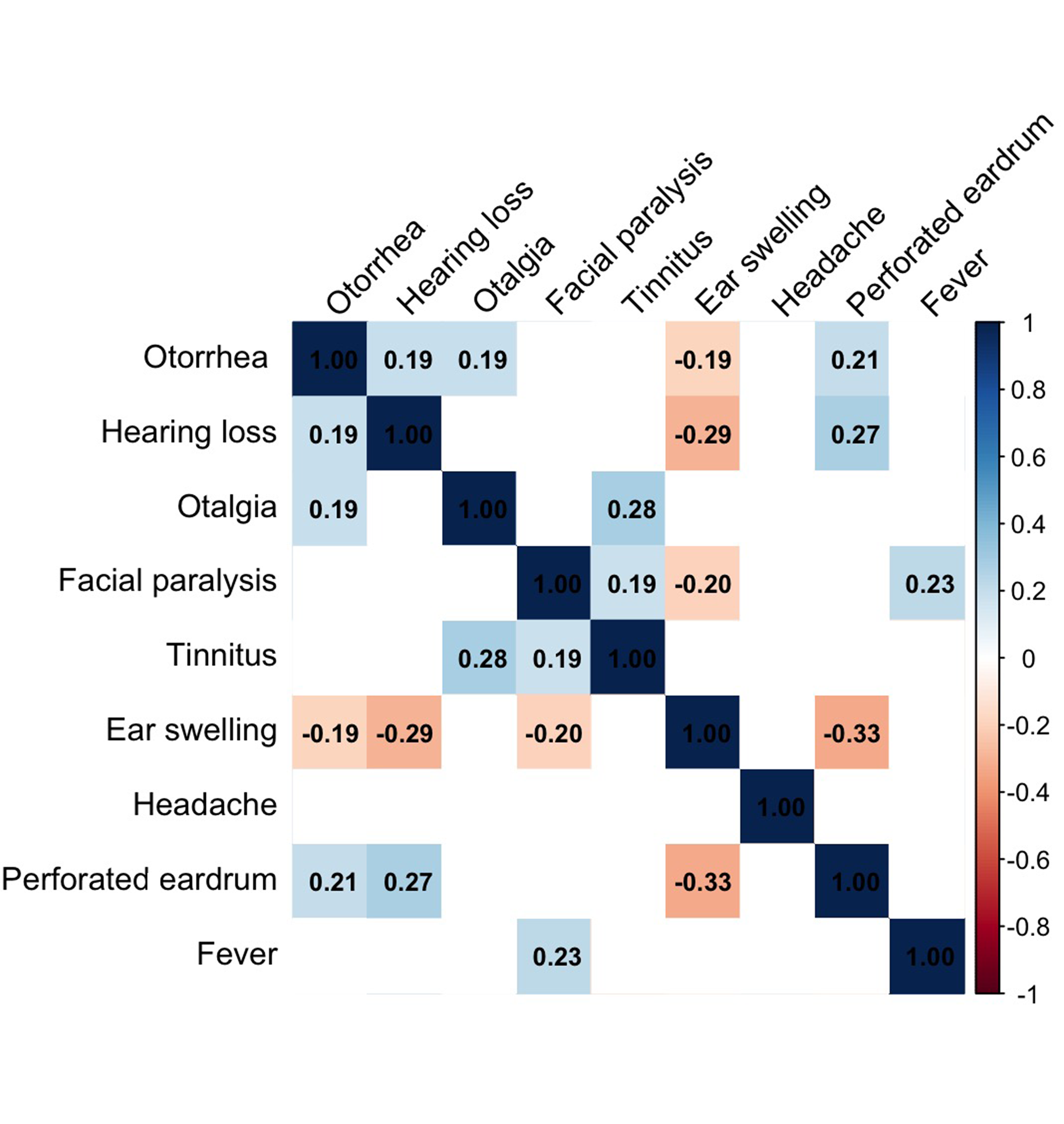

Supplement: SUPPLEMENTARY FIGURE S1 — Matrix of Pearson’s correlation coefficients among different symptoms: A color-coded correlation scale is displayed to the right of the plot. Blue represents positive correlations, while red ellipses indicate negative correlations. This heatmap includes correlations with an absolute Pearson correlation coefficient greater than 0.2 and a p-value < 0.05. [file Image_1.TIF]

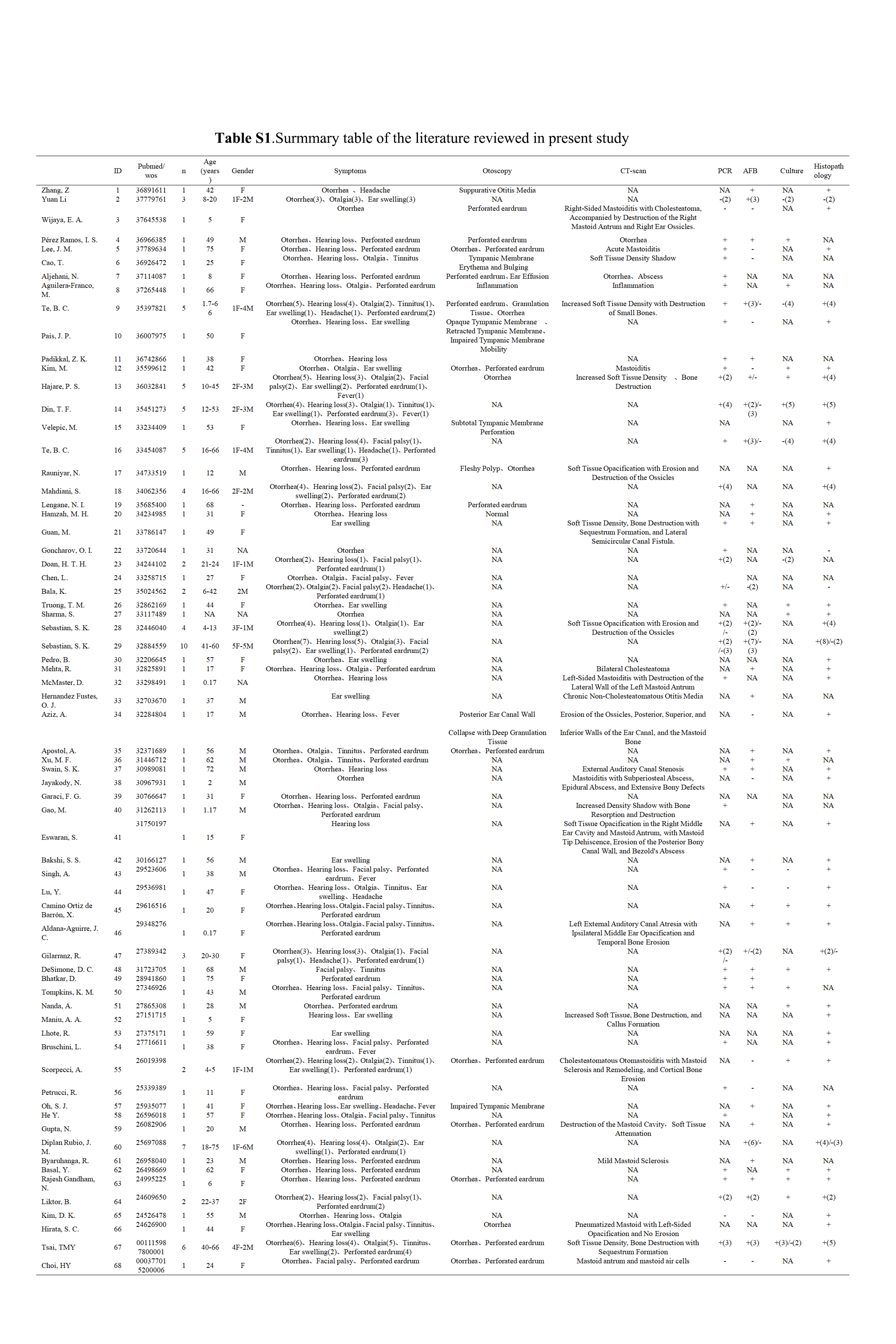

Supplement: Supplementary file 2 [file Image_2.TIF]

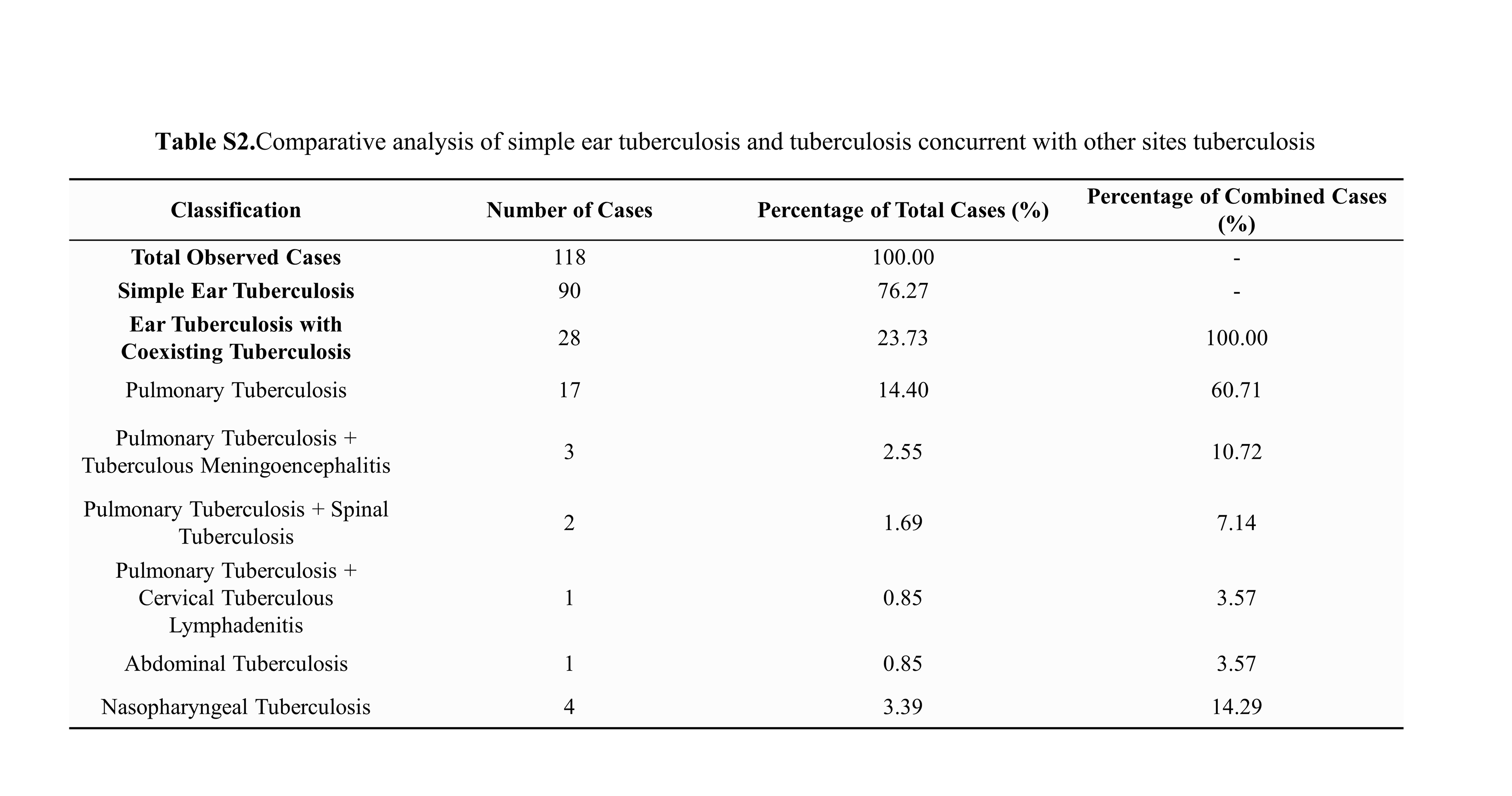

Supplement: Supplementary file 3 [file Image_3.TIF]

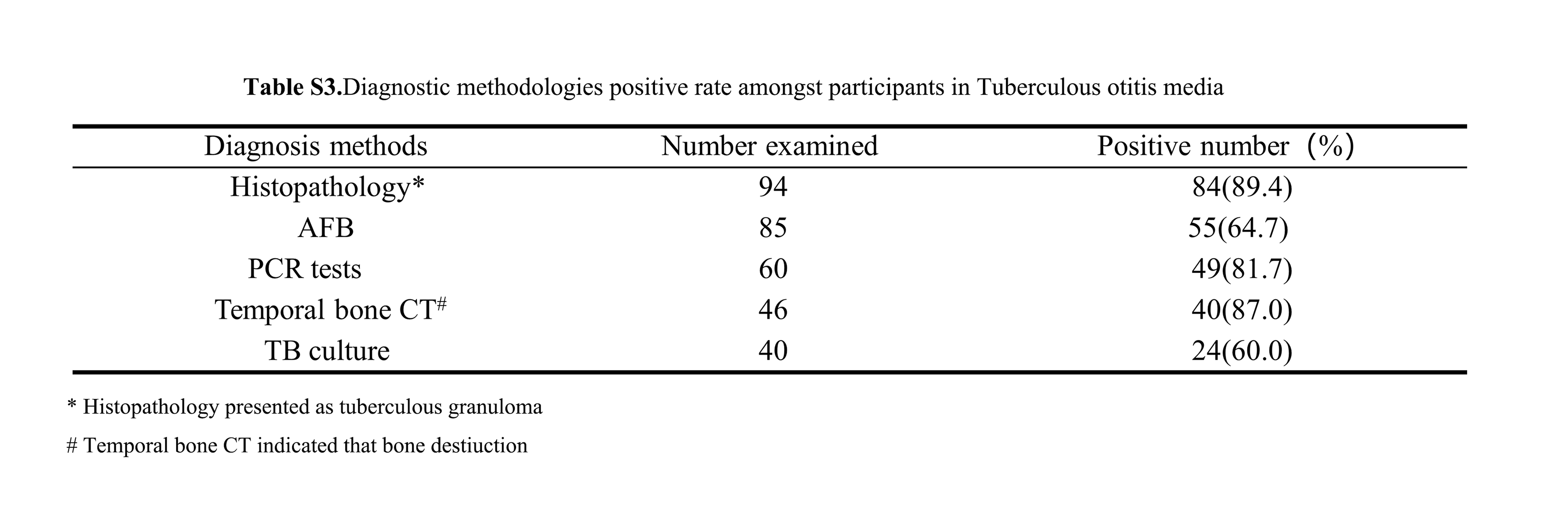

Supplement: Supplementary file 4 [file Image_4.TIF]
